# Structural insights into rice *SalTol* QTL located SALT protein

**DOI:** 10.1038/s41598-020-73517-y

**Published:** 2020-10-06

**Authors:** Navdeep Kaur, Amin Sagar, Pankaj Sharma, Ashish, Pratap Kumar Pati

**Affiliations:** 1 Department of Biotechnology, Guru Nanak Dev University, Amritsar, Punjab 143005 India; 2 CSIR-Institute of Microbial Technology, Sector 39-A, Chandigarh, 160036 India

**Keywords:** Biotechnology, Genomics, Plant biotechnology

## Abstract

Salinity is one of the major stresses affecting rice production worldwide, and various strategies are being employed to increase salt tolerance. Recently, there has been resurgence of interest to characterize *SalTol* QTL harbouring number of critical genes involved in conferring salt stress tolerance in rice. The present study reports the structure of SALT, a *SalTol* QTL encoded protein by X-ray crystallography (PDB ID: 5GVY; resolution 1.66 Å). Each SALT chain was bound to one mannose via 8 hydrogen bonds. Compared to previous structure reported for similar protein, our structure showed a buried surface area of 900 Å^2^ compared to only 240 Å^2^ for previous one. Small-angle X-ray scattering (SAXS) data analysis showed that the predominant solution shape of SALT protein in solution is also dimer characterized by a radius of gyration and maximum linear dimension of 2.1 and 6.5 nm, respectively. The SAXS profiles and modelling confirmed that the dimeric association and relative positioning in solution matched better with our crystal structure instead of previously reported structure. Together, structural/biophysical data analysis uphold a tight dimeric structure for SALT protein with one mannose bound to each protein, which remains novel to date, as previous structures indicated one sugar unit sandwiched loosely between two protein chains.

## Introduction

Salinity is one of the major abiotic stresses that significantly reduce the yield of rice^1^. In order to circumvent this problem, considerable scientific efforts for deciphering strategies for salt stress tolerance in rice are being pursued world over^2,3^. Salinity tolerance mechanism is a fairly complex process and operates at different levels^3,4^. Ion homeostasis which ensures a low Na^+^-K^+^ ratio is considered as one of the major mechanisms involved for salinity tolerance in rice^5^. Recently, a major quantitative trait loci (QTL), *SalTol* associated with the maintenance of Na^+^-K^+^ ratio that offers salinity tolerance has been mapped on chromosome 1 of rice^6,7^. The identification of *SalTol* QTL was considered as a major breakthrough in salinity tolerance breeding in rice through marker assisted backcrossing and marker assisted selection^8–10^. However, due to the lack of tightly linked markers in this region, the approach to introgress this QTL into modern high-yielding rice varieties was not that successful^11^, providing a valid ground to explore and characterize candidate genes within the *SalTol* QTL which may be critical in conferring salinity tolerance in rice.

There are a number of genes present in the *SalTol* QTL which work in a coordinated manner for achieving salt stress adaptation^7^. Among *SalTol* QTL genes of rice, *SalT* (GenBank: Z25811.1 Accession No.: Z25811.1) which encodes SALT protein (145 amino acids); a mannose binding lectin is considered crucial in governing salinity tolerance in rice^12,13^. Mannose binding proteins lectins (Man- binding lectins) are distributed across the plant kingdom and have received significant attention due to their various biological functions. They are known to possess anti-bacterial, anti-fungal, anti-viral, and anti-insecticidal properties^14–16^. In plants, the expression of Man-binding lectins is influenced by salt, drought, cold, heat, and plant growth regulators treatments^17^. Their role has also been established for plant cell protection and in stress signaling transduction^15,18^. At present, many laboratories across the world are engaged in characterization of important mannose binding genes in different plant species and trying to utilize it for crop improvement programmes^15–17,19,20^.

Till now, these Man-binding lectins have been reported and characterized from five monocot families including Liliaceae, Amaryllidaceae, Orchidaceae, Alliaceae, and Araceae^21^. However, there is scarcity of information on the characterization of Man-binding lectins from other monocotyledonous families including Poaceae. SALT is a one such glycine rich Man-binding lectin known to be induced in rice under the effect of salinity, elevated ionic concentration, sucrose starvation, and water deprivation^13,22^. The degree of the inductive response is often correlated with the amount of damage caused by the stress, Na^+^ ion accumulation, and internal ion gradient in the cell^13^. Along with *SalT* transcript expression induced under different stress conditions, its expression is also found to be regulated by developmental and hormonal clues^23,24^. These reports emphasize on the key role of SALT protein in regulation of multiple processes including plant growth, development and stress responses. Initial research efforts have allowed us to understand its regulation at transcript level in response to various treatments^13,22–24^, but no clue on the probable mechanism of its action has been deciphered.

To get better insights into the mode of action of SALT protein, the structural elucidation was considered to be important as it can facilitate insights into its functional annotation and help in answering many unanswered biological questions. However, almost from the past two decades, no work has been initiated on the structural and functional characterization of SALT protein in rice except from a recent paper which has published the structure of a rice jacalin-related lectin Orysata which is sequentially similar to SALT protein^25^. The present work delves with the detailed structural characterization of SALT protein in complex with mannose both in solution as well in crystalline state using SAXS data analysis and X-ray crystallography, respectively.

## Results

### SALT protein exists predominantly as a dimer in solution

**Figure 1:**
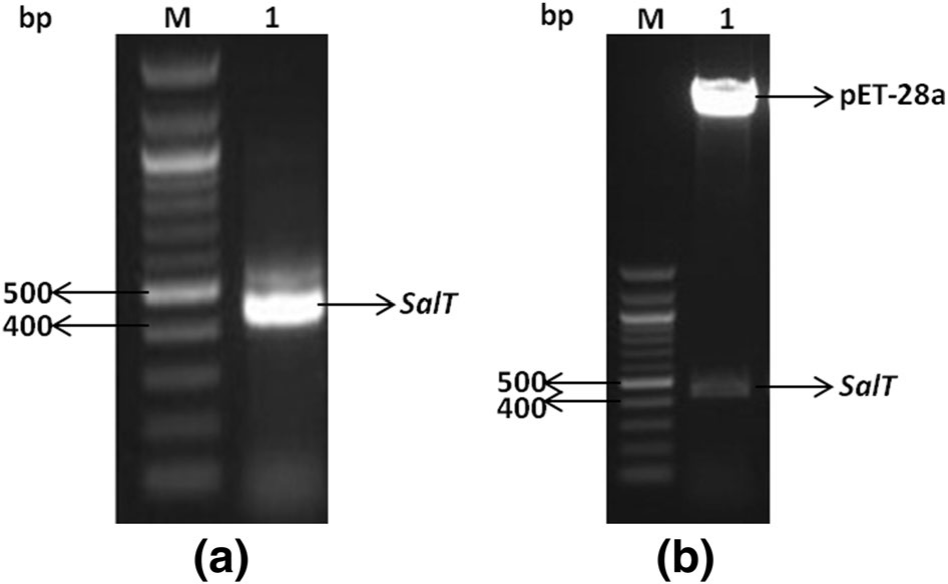
Construction of recombinant pET-28a-*SalT* expression vector. (**a**) PCR amplification of *SalT* gene. (**b**) Confirmation of *SalT* cloning in pET-28a using restriction digestion (*M* Marker).

**Figure 2:**
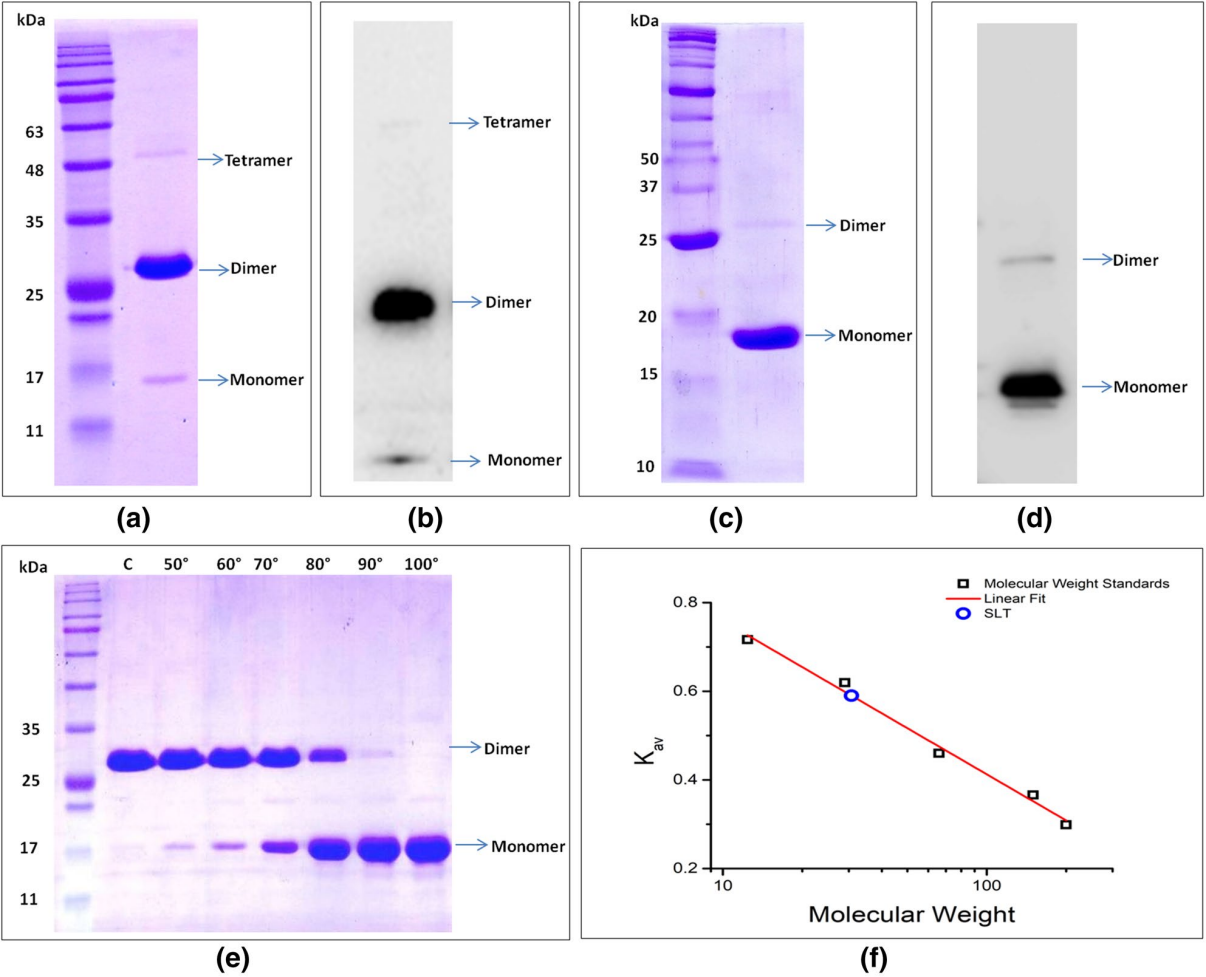
SDS–PAGE and immunoblotting analysis of SALT protein. (**a**) SDS–PAGE analysis of SALT purified protein without heat denaturation shows three bands with the most predominant dimeric band. (**b**) Immunoblotting analysis of SALT protein without heat denaturation probed with the antibody raised using the monomeric band of purified SALT. (**c**) SDS–PAGE analysis of SALT purified protein with heat denaturation at 100 °C for 5 min shows the most predominant monomeric band. (**d**) Immunoblotting analysis of heat denatured SALT protein probed with the antibody raised using the monomeric band of SALT. (**e**) Effect of temperature on the oligomeric state of SALT protein. SALT protein exists as a dimer at room temperature but onwards 50 °C the proportion of monomer band increases gradually with increase in temperature and at 100 °C it gets fully converted into monomer. (**f**) Estimation of the molecular weight of SALT using Size Exclusion Chromatography.

Full length *SalT* gene was amplified from the cDNA of *Oryza sativa* L*.* (Pusa basmati-1). Upon agarose gel electrophoresis of the amplified product, a band of about 438 bp was found that was in accord with the GenBank: Z25811.1 (Fig. 1a). The resulting product was cloned into pET-28a (+) vector and the recombinant construct was transformed into *Escherichia coli* (DH5α strain). The successful cloning of *SalT* in pET-28a vector was confirmed using restriction digestion (Fig. 1b). Recombinant plasmid isolated from the *E. coli* strain DH5α was mobilized into *E. coli* (BL21 strain) competent cells and heterologous protein expression of SALT was induced using 1 mM isopropyl-β-d-thiogalactopyranoside (IPTG). The induced protein was purified with Ni–NTA affinity chromatography kit (Thermo Fisher Scientific, USA). The SDS–PAGE analysis of the purified SALT protein showed three bands corresponding to size approximately 15, 30, and 60 kDa (Fig. 2a). However, among these 3 bands, the band corresponding to 30 kDa (dimeric form) was very prominent. When the protein was subjected to heat denaturation, the dimeric and tetrameric forms got converted into monomeric form (Fig. 2c). These results were further confirmed using immunoblotting (Fig. 2b,d). Further, SDS–PAGE analysis was conducted after heating the protein at different temperatures which brought forth that as we increased the temperature the proportion of dimer band gradually decreases and that of monomer band increases (Fig. 2e). Results were supported with the gel filtration profile of the SALT protein which primarily eluted as dimer when referenced to retention time of proteins with known apparent molecular masses (Fig. 2f). Another interesting observation was that during initial attempts of size exclusion chromatography (SEC), SALT protein did not elute at its expected time/volume from S200 column. We conjectured that this might be occurring because SALT by virtue of its mannose binding property, was binding to the carbohydrate based matrix of the S200 column. Therefore, 20 mM each of mannose and galactose were added to the protein solution as well as to the buffer to saturate the sugar binding sites of SALT and prevent it from binding to the S200 column matrix. After the addition of mannose and galactose, SALT eluted with an elution profile peaking at 16.8 ml, just before carbonic anhydrase (29 kDa) whose elution profile showed maxima at 16.9 ml. Overall, our experimental results strongly upheld that SALT protein exists primarily as a dimer in solution (Fig. 2f).

### Crystal structure of SALT protein

Co-crystallization of mannose bound SALT protein was attempted to get the atomic scale details of this protein and the residues involved in interaction with mannose. Positive hits from initial crystallization screening were further used for expansion to get good quality crystals. From crystals grown at 18 °C, we could acquire diffraction data up to 1.66 Å. The crystals were monoclinic with P12_1_1 space group with two molecules of SALT in each asymmetric unit. Crystal structure of the closest homologue available (35% sequence identity) i.e.* Helianthus tuberosus* lectin (PDB ID: 1C3M) was used as a search model to solve this structure using molecular replacement method^26^. Due to low sequence homology, initial attempts were failed to get the solution. However, after removing the side chains and considering only backbone trace, we were able to get a molecular replacement solution for our data, which solved up to R_work_ and R_free_ values of 16.18% and 20.05% with overall and last shell completeness of 98% and 99.9%, respectively (Table 1). The refined coordinates are available in the PDB database with ID 5GVY.

**Table 1:** Data collection and refinement statistics during structure determination of SALT protein at 18 °C.

Data collection	SALT protein
Resolution (Å)	50–1.66
Space group	P12_1_1
Unique reflections	31,076
**Unit cell parameters**	
a (Å)	48.29
b (Å)	58.81
c (Å)	51.56
Α	90°
Β	114.39
Γ	90°
Completeness (%)	98 (99.9)
R merge	0.03 (0.14)
Redundancy	2.5 (1.9)
Average *I/σ(I)*	35.8 (6.1)
**Refinement**	
R_work_ (%)	16.18
R_free_ (%)	20.05
Solvent content	31.33
No. of chains	A,B
**r.m.s.d. from ideality**	
Bonds (Å)	0.006
Angles (°)	0.89
Wilson B-factor (Å^2^)	18.35
**Ramachandran plot: residues**	
Most favoured (%)	88.6
Allowed region (%)	8.3
Generously allowed (%)	3.1
Disallowed (%)	0
PDB accession code	5 GVY

Values in parentheses are for the highest resolution shell. The wavelength of the X-ray beam used for data collection was 1.5418 Å.

**Figure 3:**
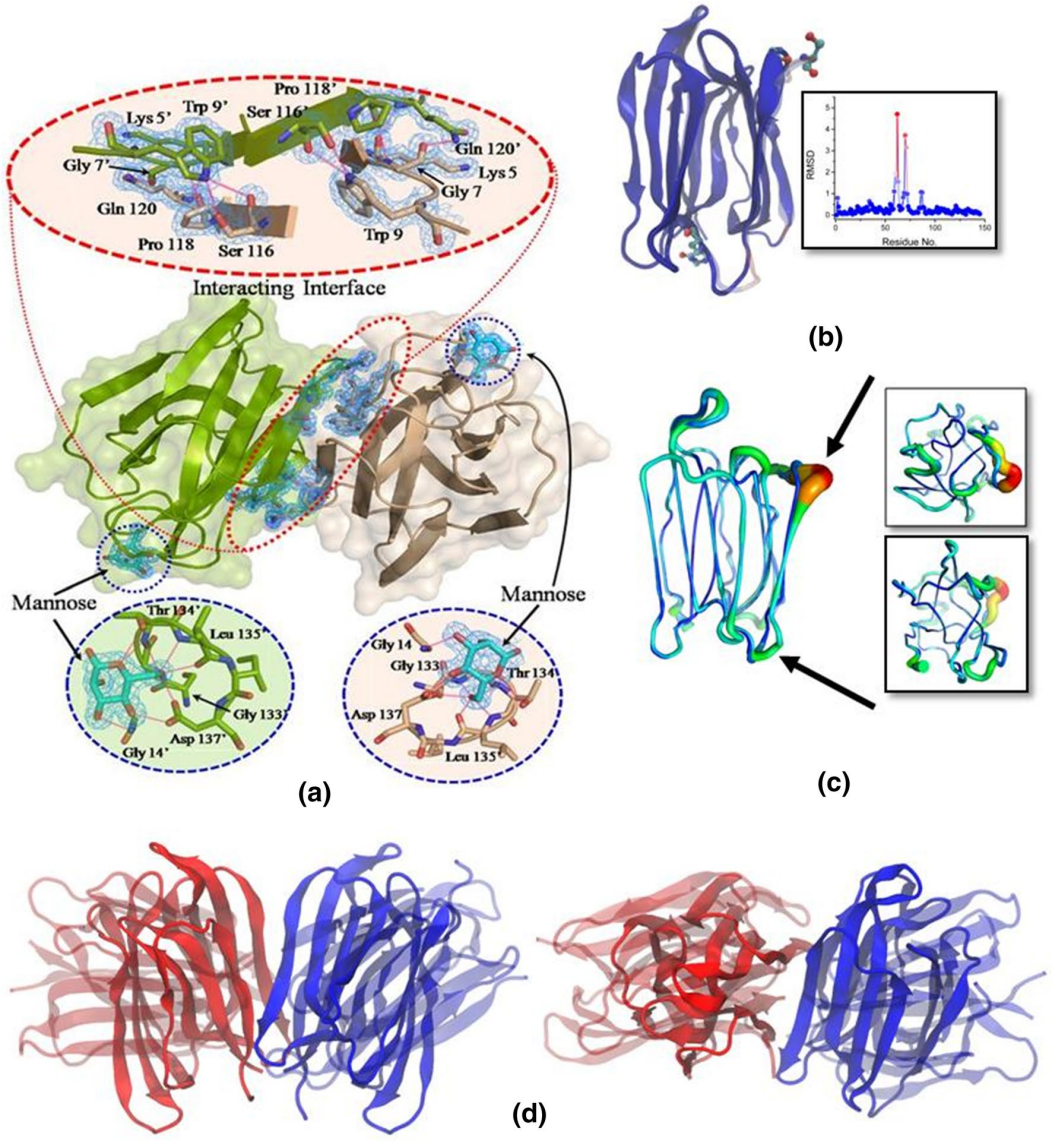
Crystal structure of SALT protein. (**a**) Crystal structure of ligand (mannose) bound SALT protein (PDB entry 5GVY). This protein exists as a dimer (Chains A and B in wheat and green colours, respectively). 2*F*_*o*_* – F*_*c*_ map of electron density at 1 σ level is shown (blue mesh) for residues involved in formation of dimeric interaction interface and ligand, mannose (coloured cyan). The insets show the zoomed in information of the residues involved in interaction interface of dimeric assembly as well as those involved in interaction with the ligand. (**b**) Structural alignment of structure reported here and PDB ID 5XFH with the residues coloured according to the RMSD from blue representing low RMSD to red representing high RMSD. The residues that differ in the two structures and are close to the high RMSD loops are shown in CPK representation. A plot of RMSD of the residues is also shown. (**c**) Similar overlay of the two structures in cartoon putty representation (radius of cartoon is proportional to β values) emphasising that the loops that differ in the orientation have much smaller β values in the structure reported here. (**d**) Two orthogonal views of the overlay of the dimeric assembly seen in the reported structure with PDB ID 5XFH.

The structure of SALT protein is predominantly composed of β-sheets (66.2%) and remaining is random coil or unstructured loops interconnecting them. There are 3 β-sheets, each composed of 4 β-strands. Interestingly, two molecules of mannose are also present in the asymmetric unit, each bound to a protein chain. Each mannose is bound to the protein through 8 H-bonds involving protein residues Gly14, Gly133, Thr134, Leu135, and Asp137 (Fig. 3a). Overall structure of the monomer is very similar to the recently solved structure of Jacalin-related lectin Orysata from *Japonica* subspecies of rice (PDB ID: 5XFH) with RMSD of 0.226^25^. The per-residue RMSD is also less than 0.5 Å for almost all the residues (Fig. 3b). The differences are mostly in the two loop regions ranging from residue 57 to 65 (Loop 1) and from 69 to 73 (Loop 2). In our structure of SALT protein, both the loops are closer to the main body of the protein and have significantly lower B-factor indicating a more stable conformation (Fig. 3c). This might be the result of the bound mannose or the two mutations i.e. G62S and H74Q, which are located in vicinity of the loops. More importantly, the dimeric assembly is completely different than the structure published earlier for Jacalin-related lectin. In case of PDB ID 5XFH, there is a very weak interaction between the two protein chains with 1 salt bridge and 10 hydrogen bonds. The buried surface area is merely ~ 240 Å^2^ which indicates that the dimeric association seen in PDB ID 5XFH is less likely to exist as dimer in solution and/or under physiological conditions, especially in the absence of the bound glycans which bridge the two protein chains. In sharp contrast, in our crystal structure of SALT protein, the interface of protein chains is stabilized through 10 hydrogen bonds and 81 hydrophobic interactions burying a surface area of ~ 900 Å^2^ supporting its existence as a stable dimer as seen in our experimental results mentioned above (Fig. 3d). Residues involved in hydrogen bonding are Lys5, Gly7, and Trp9 of one chain with Gln120′, Pro118′, and, Ser116′ of the other, involving both main chain as well as side chain residues (Fig. 3a).

### Solution shape of SALT protein

**Figure 4:**
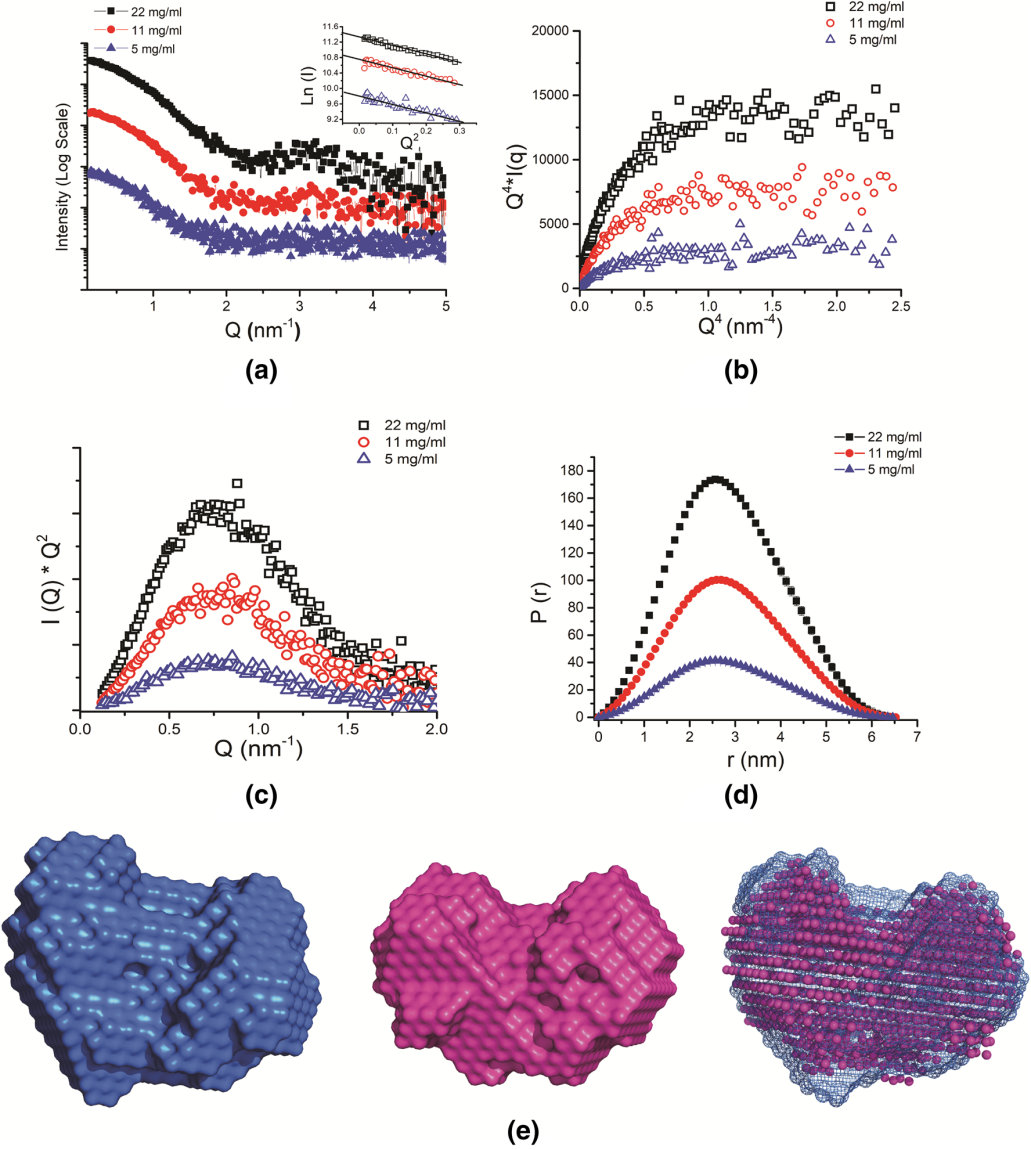
SAXS data analysis of SALT protein. (**a**) The SAXS intensity plots for SALT at three concentrations with the Guinier plots in the inset. (**b**) The corresponding Porod–Debye plot. (**c**) Kratky plots. (**d**) Distance distribution curves. (**e**) The averaged SAXS derived models assuming P1 (left) and P2 (middle) symmetries and an inertial axis overlay of the two (right).

To further confirm that the dimeric association seen in the crystal structure of SALT is the correct representation of the dimer and there are no alternative associations in space accessible to the two chains of SALT protein, we opted to acquire SAXS data for the protein and analyse the shape information *vis-a-vis* our crystal structure. SAXS Intensity datasets as function of momentum transfer vector, Q, were obtained from freshly eluted samples from gel filtration (and then concentrated) at concentrations of 5, 11 and 22 mg/ml to understand the solution shape and oligomeric state of SALT (Table S1a,b). Complete lack of any upward or downward slope in the intensity profiles or the linearity of Guinier plots presuming globular shape profile at any of the three concentrations indicated there was no aggregation or inter-particulate effect in the concentration range studied (Fig. 4a and inset). Additionally, insignificant change in the calculated Intensity value at zero angles (I_0_/C) and radius of gyration (R_g_) from Guinier approximation as a function of SALT concentration concluded that absence of protein concentration dependent alteration in scattering shape or association of protein in solution^27,28^. The R_g_ of the predominant scattering species at concentration of 5, 11 and 20 mg/ml was calculated to be 2.55 ± 0.04 nm. Further, the molecular weight of SALT protein from acquired SAXS datasets was estimated to be about 32.2 kDa using the consensus Bayesian assessment of different concentration independent measures of Molecular Weight including Porod Invariant, Volume of Correlation, size and shape of *ab-initio* reconstructed models^29^. Furthermore, using the relationship between estimated I_0_ value and protein size and concentration, well-characterized samples of lysozyme suggested molecular mass of SALT during SAXS experiments to be around 33.4 kDa. Collectively, all SAXS data analysis clearly upheld that SALT exists predominantly as a dimer in the solution. The SAXS dataset for SALT protein is available in the SASB databank with accession codes SASDGY6, SASDGZ6 and SASDG27.

To ascertain the globular folded nature of SALT protein in solution, we analysed four types of plots viz. Debye–Kratky (q^2^xI(q) *vs*. q^2^) (Fig. S1a), SIBYLS (q^3^xI(q) *vs*. q^3^) (Fig. S1b), Porod–Debye (q^4^xI(q) *vs*. q^4^) (Fig. 4b) and Kratky plot (I(q)xq^2^ *vs*. q) (Fig. 4c). The first three plots are indicative of the value of the Porod exponent with a plateau in Debye–Kratky, SIBYLS and Porod–Debye plots indicating a Porod exponent of 2, 3 and 4, respectively. We found that while Debye-Kratky and SIBYLS plot exhibited a downward slope on processing acquired SAXS datasets, the Porod–Debye plots exhibited a hyperbolic profile or plateau at higher values of Q with a Porod Exponent of 3.9 confirming compact globular scattering shape of SALT molecules^28,30^. This trend is most apparent in the SAXS data collected at 22 mg/ml because of the higher Signal-to-Noise ratio. The Kratky plots at all the concentrations had a clear peak which additionally confirmed that SALT protein dimers exist as compact globular particles in solution.

The pairwise distance distribution functions, P(r), were generated by Indirect Fourier Transformation of the intensity profiles (Fig. 4d). The real space R_g_ derived from the P(r) curves was close to 2.16 ± 0.12 nm at all concentrations. The maximum linear dimension (D_max_) was calculated to solve in the range of 6.46–6.52 nm for all the three concentrations. Since SALT protein adopts compact globular shape, its shape restoration using dummy residues in uniform density is expected to be reliable^31^. To restore scattering shape of SALT dimer from the SAXS datasets, 10 independent models were computed by DAMMIF program, aligned along their inertial axes, averaged and then further refined using DAMMIN program. This above-mentioned procedure was done first without any symmetry enforcement (P1) and then by enforcing P2 symmetry. Latter bias was used as our earlier results conclusively established that SALT exists as a dimer in the solution. The mean value of normalised spatial discrepancy (NSD) in case of 10 models generated with P1 symmetry was 0.542 with standard deviation of 0.015, while the corresponding values for the equal number of models with P2 symmetry were 0.567 and 0.036, respectively. NSD is a parameter to quantify the similarity between two or more shapes. A value of 0 implies perfectly identical shapes while a value > 1 means that the shapes are systematically different from each other. For both P1 and P2 models, the value of mean NSD for the 10 models was < 1, which indicates that the individual models were very similar to each other and that the modelling procedure is consistent and reliable. In other words, the models generated without any symmetry enforcement also have an approximate twofold symmetry. The final averaged models considering P1, P2 symmetry and their overlay is shown in Fig. 4e.

**Figure 5:**
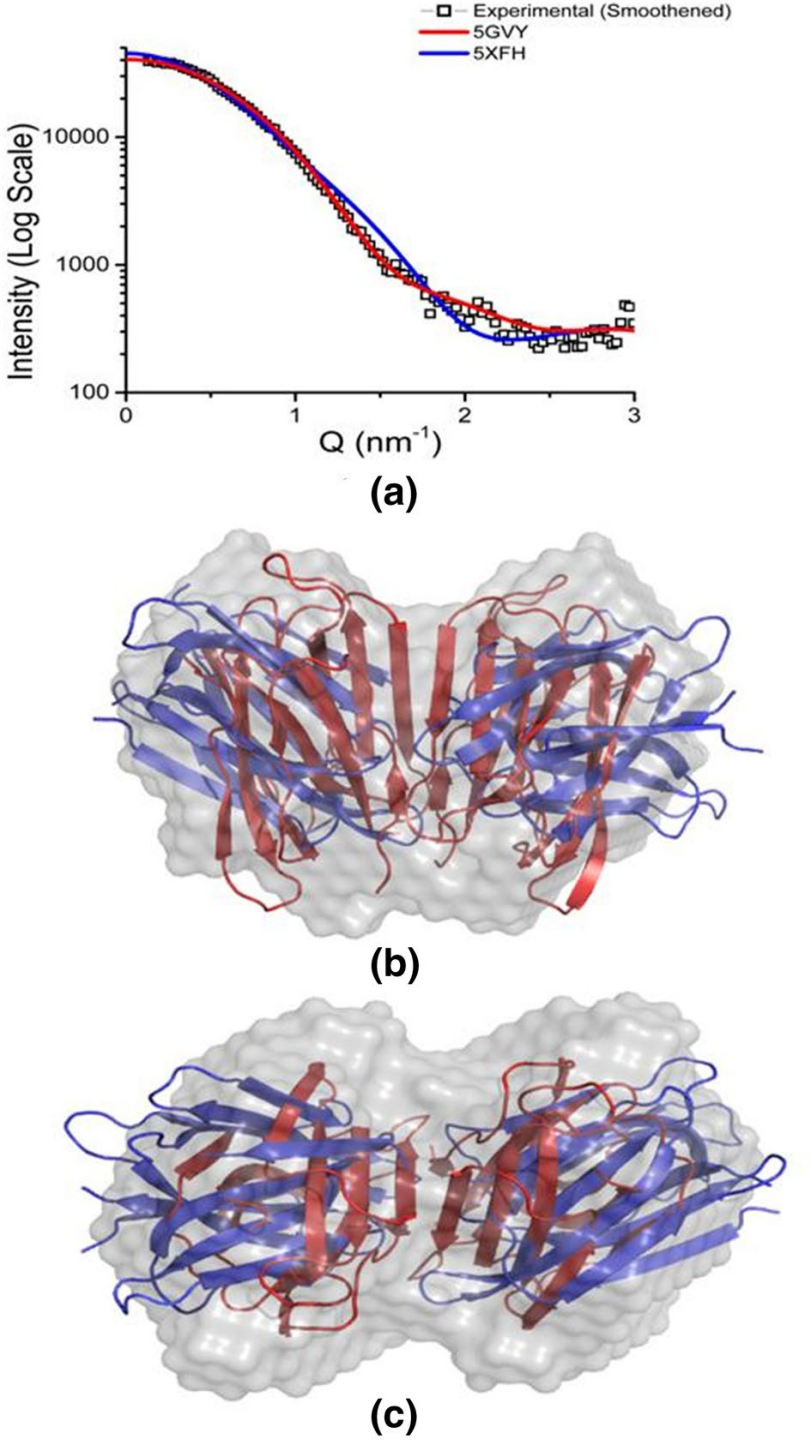
Comparative analysis of the computed SAXS profile of solved crystal structure with the experimental SAXS profile of SALT protein. (**a**) The comparison of the computed SAXS profile of the mannose bound crystal structure and structure in complex with High-Glycan (5XFH) with the experimental SAXS profile. (**b**,**c**) Two orthogonal views of the inertial axis overlay of the Mannose and High-Glycan bound structures with the averaged SAXS derived model assuming P2 symmetry.

Importantly, the computed SAXS profile of our crystal structure of SALT protein is in excellent agreement with the experimental SAXS profile with a χ^2^ value of 1.5 indicating the dimer structure seen in our crystal structure is present in solution as well (Fig. 5a). The automated superimposition of our crystal structure and that obtained in complex with higher glycans (PDB ID 5XFH) with our SAXS data-based models considering P1 and P2 symmetry are presented in Fig. 5b,c, respectively. Visually, it can be perceived that our mannose bound crystal structure of SALT protein dimer fits inside the SAXS derived envelopes much better that previous high-glycan bound structure. This was further confirmed by slightly lower NSD value of 0.54 for Mannose bound structure compared to 0.97 for High-Glycan bound structure, indicating that the dimer seen in PDB ID 5XFH might be a crystallization artifact.

## Discussion

The oligomerization state of the lectins is known to affect the binding and neutralization properties of lectins. For example, tetrameric lectins from snowdrop and daffodil bind to the glycan moieties on the HIV surface protein gp120, while the dimeric lectins from garlic don’t bind. The Jacalin-related lectins have been classified into three main categories based on their oligomeric state. The first class exists as a tetramer, e.g. Jacalin and Artocarpin, where the tetramer can be described as a dimer of dimers. The second class consists of lectins which exist as dimers e.g. Calsepa. The third class consists of tetrameric lectins which crystallise as an octamer e.g. Heltuba^29^. To understand the oligomeric state of SALT protein, we performed the different biophysical experiments. In our investigation, when the purified protein was run on a SDS–PAGE, it was found to exist in monomeric, dimeric, and tetrameric forms. Among these different forms, dimeric form was found to be most prominent. These results were further confirmed through size exclusion chromatography (SEC) analysis. Structural analysis also confirmed the dimeric status of this protein in crystalline lattice and showed that the protein is predominantly composed of β-sheets (66.2%). In the previous reports, it has been emphasized that most of the mannose binding lectins exist as homo- oligomers comprised of either two, three, or four subunits^18,32^. It is also realized that among the different oligomeric forms, dimerization of a protein is a critical factor responsible for their functional regulation in plants^33^. Dimerization provides stability to the proteins involved in numerous cellular processes^33^. Thus, the observed higher tendency of SALT protein to form a dimer could be implicated either to its flexible regulatory role or it could be an evolutionary selected feature of monocot lectins^34^. It was interesting that SALT migrated as a dimer even in SDS–PAGE gel in the presence of a detergent (SDS) and a reducing agent (BME) and conversion from dimer to monomer was found to be dependent upon temperature. The reason for this unusual stability even in standard denaturing conditions could be that differing from previous known crystal structures of similar proteins which had very low buried surface area for association and were bound via glycan moieties^25^, the interface of our SALT dimeric chains was found to be stabilized through 10 hydrogen bonds and 81 hydrophobic interactions. Thus, our results suggest that SALT exists as a very tightly bound dimer and the reason behind this could be strong hydrophobic interactions among two monomeric molecules which are stable under the denaturing and reducing conditions but disrupts as we increase the temperature.

To affirm similarity between crystal and solution shape of the SALT protein, SAXS data was acquired. Our crystal structure of SALT fitted well into the SAXS derived envelopes of SALT. Prior to us, the crystal structure of jacalin-related lectin Orysata that is almost similar to SALT showed that two monomers of Orysata can bind to one Complex-type glycan and form a 2–1 sandwich^25^. As mentioned above, this structure lacks significant buried area between protein chains and interchain interactions are mainly via sugar moiety. Comparison of SAXS data with the two crystal structures showed that SALT exists as a stable dimer, as present in the crystal structure obtained by us, where protein–protein interactions stabilize the dimeric association instead of sugar mediated interactions as present in the structure of Jacalin-related lectin Orysata from *Japonica* subspecies of rice (PDB ID: 5XFH). Overall, our studies unequivocally prove that SALT exists as a tightly bound dimer.

Finally, this is the first report on structural characterization of SALT at atomic scale and solution state. Moreover, the critical amino acid residues involved in binding of SALT to mannose have been identified which will play pivotal role in deciphering mode of action of SALT in future. Definitely, some new information is being contributed to this area of study, yet many queries remain, particularly functional relevance of the dimeric state and high number of H-bonds holding the mannose molecule. In simplistic terms, it can be speculated that the strong binding between SALT with mannose is likely to contribute in its role in salt stress tolerance. The accumulation of reducing sugars in cell cytosol is known to provide osmoprotection in plants in response to abiotic stress stimuli^35^. As mannose is a reducing sugar, it is hypothesized that build-up of mannose in bound form with SALT inside the cell will result in increased cellular osmotic potential and thereby confers salinity stress tolerance to plants. However, in normal situation, the levels of free mannose are very low in the cell cytoplasm due to its fast metabolism which offsets our hypothesis^36^. Many of these pending queries can be addressed in future by taking insights from the structural analysis of SALT protein by our group and other researchers in the field.

## Methodology

### Construction of recombinant pET-28a-*SalT* expression vector

Full length *SalT* gene was amplified from the cDNA of *Oryza sativa* L*.* (Pusa basmati-1) using a pair of full length gene specific primers (forward: atgGGATCCATGACGCTGGTGAAGATTGG;reverse:ctGAGCTCTCAAGGGTGGACGTAGATGC). The amplified fragment was ligated in the protein expression vector pET-28a (+) using two restriction enzymes, BamHI and SacI under the control of an inducible promoter lac operon. The recombinant construct was transformed into *E. coli* (DH5α strain) cells. Cloning was confirmed using restriction enzyme digestion and the recombinant plasmid purified from *E. coli* strain DH5α was then mobilized into *E. coli* (BL21 DE3 strain) competent cells.

### Protein expression and purification

The *SalT* gene (Genbank Accession No.: Z25811.1) cloned in protein expression vector pET-28a (+) was used for heterologously expressing the SALT protein in *E. coli* (BL21 DE3 strain) at 37 °C with 1 mM isopropyl-β-d-thiogalactopyranoside (IPTG). The bacterial proteins were extracted by lysing the cells using 50 mM sodium phosphate buffer, pH 8.0, containing 0.3 mM NaCl and 10 mM imidazole followed by sonication for 5 min on ice (9 s on and 5 s off at 40% amplitude). The homogenate was then centrifuged at 12,000*g* for 20 min at 4 °C, and the protein present in the supernatant was purified using the Ni–NTA affinity chromatography kit (Thermo Fisher Scientific, USA). The column was equilibrated using PBS + 10 mM imidazole before protein binding and was later washed using PBS + 40 mM imidazole. The purified protein was eluted with PBS + 250 mM imidazole.

### SDS–PAGE analysis of purified SALT protein

SDS–PAGE analysis of the heterologously purified SALT protein was carried out as per the Laemmli (1970) method^37^ in mini protein gel running apparatus (Bio-Rad, USA). SALT protein (1 mg/ml) was loaded on 12% resolving and 5% stacking gel both after heat denaturing the protein at 95 °C for 5 min and without heat denaturation. Further, for analysis of the effect of temperature on the different oligomeric forms of SALT protein, it was heat denatured at different temperatures (50 °C, 60 °C, 70 °C, 80 °C, 90 °C, and 100 °C) for 5 min each before cooling to room temperature and was loaded on SDS–PAGE gel. The electrophoresis was carried out at 80 V for stacking and at 100 V for resolving using 1X running buffer (18.8 glycine, 3.02 tris, and 1 g SDS per 1 L). After running, the gels were dipped in Coomassie brilliant blue staining solution overnight (0.1% Coomassie brilliant blue –R, 50% methanol, 10% acetic acid and 40% water). The gels were then destained using 20% methanol, 10% acetic acid, and 70% water. The images were captured using the Bio-Rad’s Gel Doc Imager XR + system.

### Immunoblotting of SALT protein

The primary antibody against the SALT monomeric protein band was used for the immunoblotting (custom made; rabbit polyclonal, GeNei, cat. no. 640501100011730). 1 mg/ml of SALT protein was run on 12% SDS–PAGE resolving gel. After running, the gel was transferred to the PVDF membrane using mini protein transfer unit (Bio-Rad, USA). After transfer, the membrane was blocked using 5% skimmed milk for 1 h and then was probed with 1° antibody prepared in 5% skimmed milk in 1:1000 ratio at 4 °C overnight. The membrane was washed 4 times using 1X TBST buffer and was probed with HRP conjugated 2°antibody (Abcam, UK, ab6721) raised in goat using the desired dilution of 1:3000. After washing 4 times with 1X TBST buffer, blot was developed using commercial ECL solution.

### Size exclusion chromatography

The purified SALT obtained after Ni-affinity chromatography was concentrated to 5 mg/ml using membrane concentrators with cut-off of 3 kDa (Millipore). 200 µl of the concentrated protein was injected into a Superdex 200 10/300 GL column attached to an ÄKTA Explorer FPLC (GE Biosciences) after the addition of 20 mM each of mannose and galactose. The running buffer had the same composition as the dialysis buffer except that it was supplemented with 20 mM each of mannose and galactose. The column was calibrated using gel filtration markers kit (Sigma) consisting of Cytochrome c (12.4 kDa), Carbonic Anhydrase (29 kDa), Albumin (66 kDa), Alcohol Dehydrogenase (150 kDa), β-Amylase (200 kDa), and Blue dextran (2000 kDa). The data was analyzed using the Origin 7 software.

### Crystallization and X-ray diffraction data collection

SALT protein was purified and concentrated to 17 mg/ml for crystallization set-ups. Initially, crystallization screening kits were used to get the crystals, 1:1 ratio of protein to reservoir solution was used in crystallization drops. Screens from Molecular Dimensions (Structure Screen, PACT primer), Hampton Research (Crystal Screen, Index), and Wizard Screen (Emerald Bio) were used. Crystallization plates were kept in vibration free RUMED unit maintained at 18 °C. Diffractable, but multi-lattice crystals were observed under many conditions after one week. However, few single crystals were also formed in some conditions. Single crystals were then used for diffraction quality assessment and we successfully obtained data up to 3 Å in a couple of conditions. These conditions were further expanded to get better quality diffraction data. Finally, diffraction data at 1.66 Å resolution was obtained from crystals grown in reservoir solution having 200 mM NaCl, 100 mM Tris pH 8.8, and 25% w/v PEG 3350. The crystals were diffracted on in-house RIGAKU MicroMax-007HF instrument^38^. Diffraction data was collected under cryo-conditions at 100 K and crystals were prior soaked in reservoir solution for cryo-protection (since it was composed of 25% PEG 3350). Sample to detector distance was 120 mm. Each frame was collected for 10 min with 1° oscillation. Diffraction data processing including intensity integration and scaling was done using HKL2000^39^.

### Structure refinement

The number of chains in the asymmetric unit and space group was determined with Matthews Coefficient and POINTLESS programs of CCP4i suite, respectively^40,41^. Sequence identity of SALT protein was ~ 35% to the closest homologue structure (PDB id :1C3M) present in Protein Data Bank^26^. Due to low sequence identity with search model (1C3M), initial trials of molecular replacements were unsuccessful. Side chains of the search model were then truncated using CHAINSAW program of CCP4i suit^42^. The truncated model was then used as search model along with sequence of SALT protein for PHENIX AUTOSOL and AUTOBUILD wizards to build the correct structure of this protein^43,44^. COOT and phenix refine programs were used for further refinement and model building till we got complete model^45,46^. Solvent molecules were added once R_w_ value reached around 0.25 and F_o_ – F_c_ map had more than 3σ value above the mean, also forming at least one hydrogen bond with protein or other solvent atom. PROCHECK was used to validation of the refined models^47^.

### SAXS data collection and analysis

All the SAXS data reported in this study was acquired using SAXSpace instrument (Anton Paar GmbH, Austria). The instrument has a line-collimated X-ray beam with wavelength of 0.154 nm. The sample to detector distance was 317.6 mm and the scattered X-rays were recorded on a 1D CMOS MYTHEN detector (Dectris, Switzerland). The SAXS data was collected at three concentrations of SALT protein (5, 11 and 22 mg/ml). For each concentration of the protein and the matched buffer, three frames of 10 min each were collected in a thermostated quartz capillary with a diameter of 1 mm maintained at 20 °C. The data was calibrated for the beam position using SAXStreat software. The SAXSquant software was then used for buffer subtraction, desmearing and binning of data within the usable angular range. The SAXS data was further analyzed using the programs available in the ATSAS 2.7.2 suite^48^. The radius of gyration (R_g_) was calculated on the basis of Guinier approximation using the program PRIMUS^49^. The distance distribution function was then calculated using the program GNOM which performs an Indirect Fourier transformation on the SAXS intensity profile^50^. The molecular weight was calculated using the program DATMOW. Ten independent ab-initio models were generated using the program DAMMIF^51^, averaged and filtered using the DAMAVER suite^52^ and refined using the program DAMMIN^51^. The modelling was done considering both P1 and P2 symmetries. The final model obtained from DAMMINs^53^ was superimposed over the high resolution structures using the program SUPCOMB^54^.

## Supplementary information

Supplementary file 1

## References

[1] Li Z (2017). Characterization of salt-induced epigenetic segregation by genome-wide loss of heterozygosity and its association with salt tolerance in rice (*Oryza sativa* L.). Front. Plant Sci..

[2] Ismail AM, Horie T (2017). Molecular breeding approaches for improving salt tolerance. Annu. Rev. Plant Biol..

[3] Kaur N, Pati PK (2017). Integrating classical with emerging concepts for better understanding of salinity stress tolerance mechanisms in rice. Front. Environ. Sci..

[4] Negr S, Schmo SM (2017). Evaluating physiological responses of plants to salinity stress. Ann. Bot..

[5] Munns R, Tester M (2008). Mechanisms of salinity tolerance. Annu. Rev. Plant Biol..

[6] Platten JD, Egdane JA, Ismail AM (2013). Salinity tolerance, Na + exclusion and allele mining of HKT1; 5 in *Oryza sativa* and *O. glaberrima*: many sources, many genes, one mechanism ?. BMC Plant Biol..

[7] Waziri A, Kumar A, Purty R (2016). Saltol QTL and their role in salinity tolerance in rice. Austin J. Biotechnol. Bioeng..

[8] Thi L, Huyen N, Cuc LM, Ham LH, Khanh TD (2013). Introgression the SALTOL QTL into Q5DB, the elite variety of Vietnam using marker-assisted-selection (MAS). Am. J. Biosci..

[9] Ali S (2013). Stress indices and selectable traits in SALTOL QTL introgressed rice genotypes for reproductive stage tolerance to sodicity and salinity stresses. F. Crop. Res..

[10] Babu NN, Krishnan SG, Vinod KK, Krishnamurthy SL, Schranz ME (2017). Marker aided incorporation of saltol, a major QTL associated with seedling stage salt tolerance, into *Oryza sativa* ‘Pusa Basmati 1121’. Front. Plant Sci..

[11] Chattopadhyay K (2014). Diversity and validation of microsatellite markers in Saltol QTL region in contrasting rice genotypes for salt tolerance at the early vegetative stage. Aust. J. Crop Sci..

[12] Ganie SA, Karmakar J, Roychowdhury R, Mondal TK, Dey N (2014). Assessment of genetic diversity in salt-tolerant rice and its wild relatives for ten SSR loci and one allele mining primer of salT gene located on 1st chromosome. Plant Syst. Evol..

[13] Claes B (1990). Characterization of a rice gene showing organ-specific expression in response to salt stress and drought. Plant Cell.

[14] Coumou J (2019). The role of mannose binding lectin in the immune response against *Borrelia burgdorferi* sensu lato. Sci. Rep..

[15] Barre A, Bourne Y, Van Damme EJM, Rougé P (2019). Overview of the structure: function relationships of mannose-specific lectins from plants, Algae and Fungi. Int. J. Mol. Sci..

[16] George BS, Silambarasan S, Senthil K, Jacob JP, Ghosh Dasgupta M (2018). Characterization of an insecticidal protein from *Withania somnifera* against Lepidopteran and Hemipteran pest. Mol. Biotechnol..

[17] He X (2017). A rice jacalin-related mannose-binding lectin gene, OsJRL, enhances *Escherichia coli* viability under high salinity stress and improves salinity tolerance of rice. Plant Biol..

[18] De Hoff PL, Brill LM, Hirsch AM (2009). Plant lectins: the ties that bind in root symbiosis and plant defense. Mol. Genet. Genomics.

[19] Duan X (2018). Expression of Pinellia pedatisecta lectin gene in transgenic wheat enhances resistance to wheat aphids. Molecules.

[20] Hemantaranjan, A. Molecular physiology of abiotic stresses in plant productivity. (Scientific Publishers, 2018).

[21] Hu Z (2005). Structural mechanism governing the quaternary organization of monocot mannose-binding lectin revealed by the novel monomeric structure of an orchid lectin. J. Biol. Chem..

[22] Tseng T, Tsai T, Lue M, Lee H (1995). Identification of sucrose-regulated differential display genes in cultured rice cells using mRNA SRl. Genes.

[23] Garcia AB, Engler JA, Claes B, Villarroel R, Van Montagu M, Gerats T, Caplan A (1998). The expression of the salt-responsive gene salT from rice is regulated by hormonal and developmental cues. Planta.

[24] Souza AD (2003). Accumulation of SALT protein in rice plants as a response to environmental stresses. Plant Sci.

[25] Nagae M, Mishra SK, Hanashima S, Tateno H, Yamaguchi Y (2017). Distinct roles for each N-glycan branch interacting with mannose-binding type Jacalin-related lectins Orysata and Calsepa. Glycobiology.

[26] Bourne Y (1999). Helianthus tuberosus lectin reveals a widespread scaffold for mannose-binding lectins. Strcture.

[27] Ashish (2007). Global structure changes associated with Ca2+ activation of full-length human plasma gelsolin. J. Biol. Chem..

[28] Sagar A, Haleem N, Bashir YM, Ashish A (2017). Search for non-lactam inhibitors of mtb β-lactamase led to its open shape in apo state: new concept for antibiotic design. Sci. Rep..

[29] Akkouh O (2015). Lectins with anti-HIV activity: a review. Molecules.

[30] Rambo RP, Tainer JA (2011). Characterizing flexible and intrinsically unstructured biological macromolecules by SAS using the Porod-Debye law. Biopolymers.

[31] Pandey K (2014). Low pH overrides the need of calcium ions for the shape-function relationship of calmodulin: resolving prevailing debates. J. Phys. Chem. B.

[32] Barre A, Van Damme EJM, Peumans WJ, Rougé P (1996). Structure-function relationship of monocot mannose-binding lectins. Plant Physiol..

[33] Marianayagam NJ, Sunde M, Matthews JM (2006). The power of two: protein dimerization in biology. Trends Biochem. Sci..

[34] Klemm JD, Schreiber SL, Crabtree GR (1998). Dimerization as a regulatory mechanism in signal transduction. Annu. Rev. Immunol..

[35] Sami F, Yusuf M, Faizan M, Faraz A, Hayat S (2016). Role of sugars under abiotic stress. Plant Physiol. Biochem..

[36] Hu X, Shi Y, Zhang P, Miao M, Zhang T, Jiang B (2016). D-Mannose: properties, production, and applications: an overview. Comprehensive Rev. Food Sci. Food Saf..

[37] Keller A, Pedemonte E, Willmouth FM (1970). © 1970 nature publishing group. Nature.

[38] Tomar R, Bansal S, Kundu B (2014). Structural and functional insights into an archaeal l-asparaginase obtained through the linker-less assembly of constituent domains. Acta Crystallograph. Sect. D Biol. Crystallograph..

[39] Minor W, Otwinowski Z (1997). Processing of X-ray diffraction data collected in oscillation mode. Methods Enzymol..

[40] Kantardjieff KA (2003). Matthews coefficient probabilities:improved estimates for unit cell contents of proteins, DNA, and protein: nucleic acid complex crystals. Protein Sci..

[41] Evans PR (2011). An introduction to data reduction: space-group determination, scaling and intensity statistics. Crystallograph. Sect. D Biol. Crystallograph.

[42] Stein N (2008). CHAINSAW: a program for mutating pdb files used as templates in molecular replacement. J. Appl. Crystallograph..

[43] Pavel V (2008). Iterative model building, structure refinement and density modification with the PHENIX AutoBuild wizard. Acta Crystallograph. Sect. D Biol. Crystallograph..

[44] Read RJ, Airlie J (2009). Decision-making in structure solution using Bayesian estimates of map quality: the PHENIX AutoSol wizard. Acta Crystallograph. Sect. D Biol. Crystallograph..

[45] Emsley P, Cowtan K (2004). Coot: model-building tools for molecular graphics. Acta Crystallograph. Sect. D Biol. Crystallograph..

[46] Afonine PV, Ralf W, Headd JJ, Thomas C (2012). Towards automated crystallographic structure refinement with phenixrefine. Acta Crystallograph. Sect. D Biol. Crystallograph.

[47] Laskowski RA, Moss DS, Thornton JM (1993). Main-chain bond lengths and bond angles in protein structures. J. Mol. Biol..

[48] Petoukhov MV (2012). New developments in the ATSAS program package for small-angle scattering data analysis. J. Appl. Crystallograph..

[49] Konarev PV, Volkov VV, Sokolova AV, Koch HJ, Svergun DI (2003). PRIMUS: a Windows PC-based system for small- angle scattering data analysis. J. Appl. Crystallograph.

[50] Svergun DI (1992). Determination of the regularization parameter in indirect-transform methods using perceptual criteria. J. Appl. Crystallogr..

[51] Svergun DI (1999). Restoring low resolution structure of biological macromolecules from solution scattering using simulated annealing. Biophys. J..

[52] Volkov VV, Svergun DI (2003). Uniqueness of ab initio shape determination in small-angle scattering. J. Appl. Crystallogr..

[53] Franke D, Svergun DI (2009). DAMMIF, a program for rapid ab-initio shape determination in small-angle scattering. J. Appl. Crystallogr..

[54] Kozin MB, Svergun DI (2001). Automated matching of high- and low-resolution structural models. J. Appl. Crystallograph..

