# Supplementary material for: Structural insights into rice SalTol QTL located SALT protein
Source: Sci Rep. 2020 Oct 6;10:16589. doi: 10.1038/s41598-020-73517-y (PMC7538587; doi:10.1038/s41598-020-73517-y)
Supplement: Supplementary file 1 [file 41598_2020_73517_MOESM1_ESM.pdf]

Structural Insights into Rice *SalTol* QTL Located SALT Protein

Navdeep Kaur[1§], Amin Sagar[2§], Pankaj Sharma[2§], Ashish [2*] and Pratap Kumar Pati[1*]

[1]Department of Biotechnology, Guru Nanak Dev University, Amritsar-143005 (Punjab), India.

[2]CSIR-Institute of Microbial Technology, Chandigarh, 160036 India.

[§]These authors contributed equally in this work.

E-mail addresses:

Navdeep Kaur: kaurnavdeep302@yahoo.com; Amin Sagar: aamin.sagar@gmail.com; Pankaj Sharma: pankajsharmaimt@gmail.com; Ashish: ashgang@imtech.res.in.

*Correspondence

Dr. Pratap Kumar Pati,

Department of Biotechnology, Guru Nanak Dev University, Amritsar-143005 (Punjab), India.

E-mail: pkpati@yahoo.com, Tel: +91-183-2255802-09 Ext No. 3177, Fax: +91-183-2258272.

Dr. Ashish,

CSIR Institute of Microbial Technology, Sector 39-A, Chandigarh 160036, India.

E-mail: ashgang@imtech.res.in.

Supplementary Figure S1

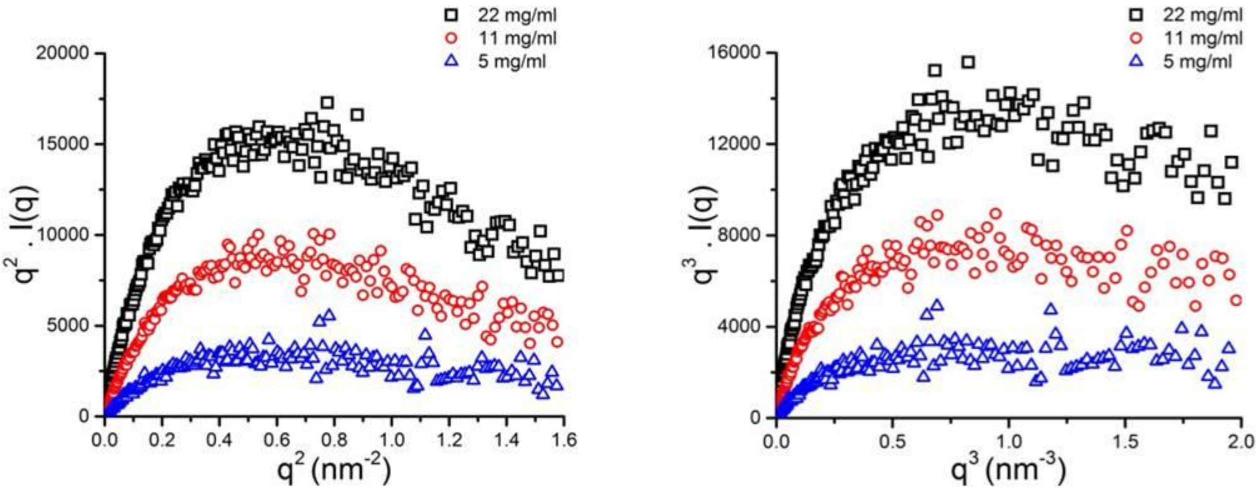

(a) **(b)**

Figure S1. SAXS analysis of SALT protein. (a) Debye-Kratky. (b) SIBYLS SAXS intensity plots for SALT protein at three concentrations.

Supplementary Tables

Table S1: Data-collection and scattering-derived parameters.

Supplementary Table S1a

**Data-collection parameters**	
Instrument /	SAXSpace (Anton Paar)
Beam geometry	10 mm slit
Wavelength (Å)	1.5418
q range (Å^{-1})	0.010–0.300
Temperature (K)	283
**Molecular-mass determination†**	
Partial specific volume (cm^3 g^{-1})	0.724
Calculated monomeric M_r from sequence	15440
Dry volume calculated from sequence (Å^3)	18710
**Software employed**	
Primary data reduction	*SAXSquant*
Data processing	*PRIMUS QT*
Ab initio analysis	*DAMMIF/DAMMIN*
Validation and averaging	*DAMAVER*
Rigid-body modelling	N/A
Computation of model intensities	*CRYSOL*
Three-dimensional graphics representations	*PyMOL*

Supplementary Table S1b

	SALT		
**Data-collection parameters**			
**Exposure time (min)**	30	30	30
**Concentration range (mg ml^{-1})**	5	11	22
**Structural parameters†**			
***I(0)* (cm^{-1}) [from *P(r)*]**	16230 ± 341	31330 ± 493	83690 ± 584
**R_g (Å) [from *P(r)*]**	2.52 ± 0.18	2.50 ± 0.13	2.55 ± 0.04
***I(0)* (cm^{-1}) (from Guinier)**	16783 ± 376	32071 ± 403	84849 ± 601
**R_g(Å) (from Guinier)**	2.50 ± 0.14	2.56 ± 0.12	2.55 ± 0.09
**D_{max} (nm)**	6.46	6.47	6.52
**Molecular-mass determination**			
**Molecular mass M_r [from V_c]**	33.6	33.5	32.2

Figure 1a

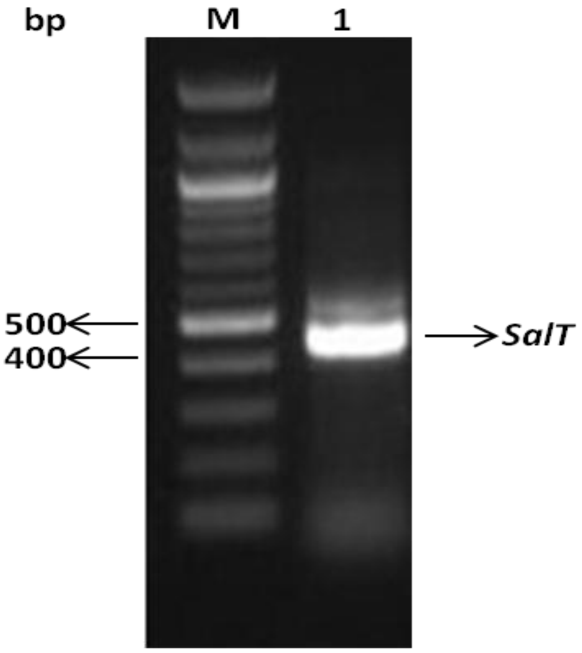

Figure 1a. PCR amplification of *SalT* gene. (M: Marker).

.

Figure 1b

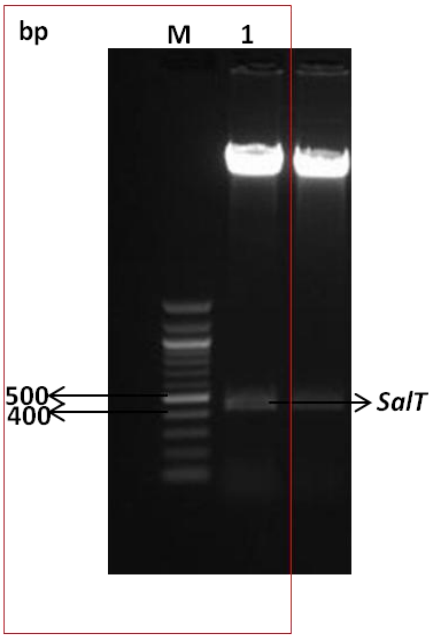

Figure 1b. Confirmation of *SalT* cloning in pET-28a using restriction digestion. (M: Marker).

Figure 2a

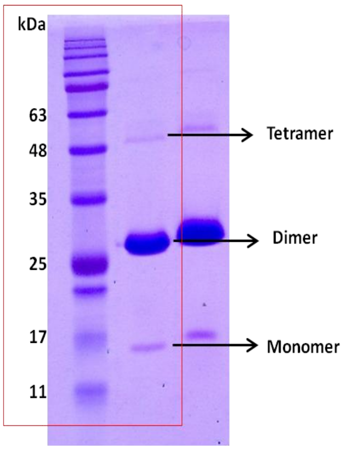

Figure 2a. SDS-PAGE analysis of SALT purified protein without heat denaturation shows three bands with the most predominant dimeric band.

Figure 2b

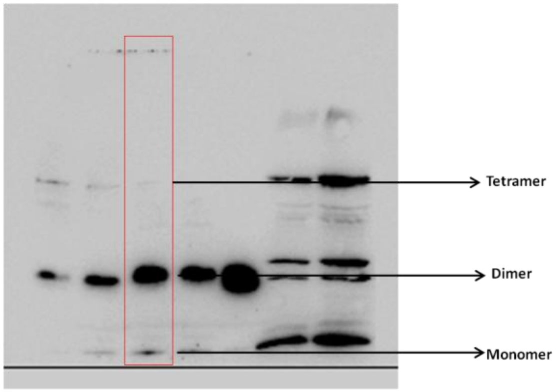

Figure 2b. Immunoblotting analysis of SALT protein without heat denaturation probed with the antibody raised using the monomeric band of purified SALT.

Figure 2c

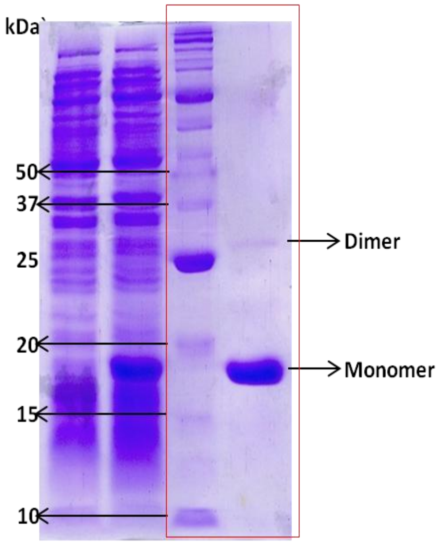

Figure 2c. SDS-PAGE analysis of SALT purified protein with heat denaturation at 100°C for 5 minutes shows the most predominant monomeric band.

Figure 2d

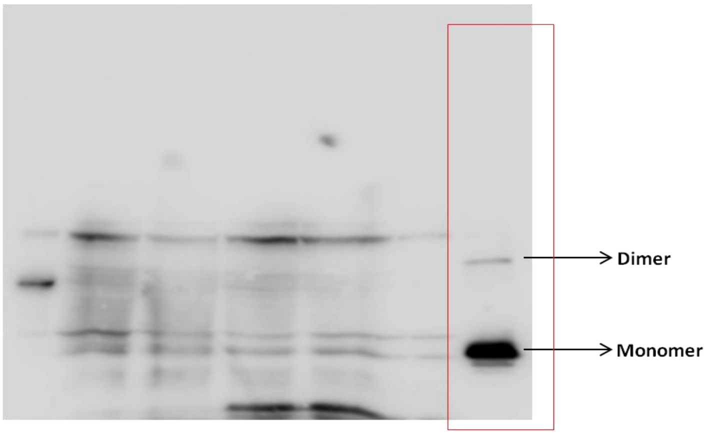

Figure 2d. Immunoblotting analysis of heat denatured SALT protein probed with the antibody raised using the monomeric band of SALT.

Figure 2e

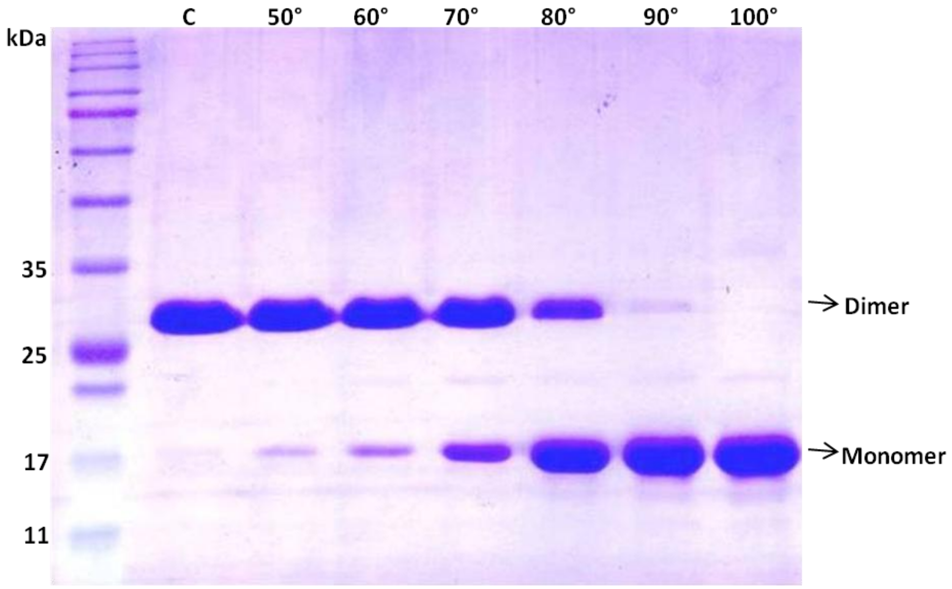

Figure 2e. Effect of temperature on the oligomeric state of SALT protein. SALT protein exists as a dimer at room temperature but onwards 50° C the proportion of monomer band increases gradually with increase in temperature and at 100°C it gets fully converted into monomer.
